# Does the law matter? An empirical study on the accessibility of Finnish higher education institutions’ web pages

**DOI:** 10.1007/s10209-022-00931-6

**Published:** 2022-11-14

**Authors:** Merja Laamanen, Tarja Ladonlahti, Hannu Puupponen, Tommi Kärkkäinen

**Affiliations:** 1 Faculty of Information Technology, University of Jyväskylä, Box 35, 40014 Jyväskylä, Finland; 2 Faculty of Education and Psychology, University of Jyväskylä, Jyväskylä, Finland; 3 University of Jyväskylä, Jyväskylä, Finland

**Keywords:** Web accessibility, Web accessibility evaluation tools, Higher education, Web content accessibility guidelines (WCAG), Students with special educational needs and disabilities

## Abstract

Information and communication technology (ICT) has made higher education available to many students in a new way. The role of online learning in higher education institutions (HEIs) has grown to an unprecedented scale due to the COVID-19 pandemic. The diversity of higher education students has increased, and accessible solutions are needed. New European and national regulations support these trends. The research reported in this paper was conducted in Finland, which is one of the leading European countries in terms of high technology and digitalisation. The aim of this research is to explore the accessibility of all Finnish HEIs’ (*N* = 38) landing pages based on Web Content Accessibility Guidelines (WCAG 2.1). The situations before and after recent legislation are compared. Previous studies have shown that HEIs’ landing pages typically have many accessibility errors. Unlike previous studies, this study considered the types of accessibility errors at a detailed level to support HEIs’ development and implementation of accessibility standards. A combination of two automated accessibility testing tools was used, and the performance of individual tools was analysed. The results show that HEIs’ landing pages are not accessible and there are enormous differences between institutions. Two clusters of HEIs were found: one with good accessibility in terms of WCAG 2.1’s four principles (perceivable, operable, understandable, and robust), and one with poor accessibility. On half of the HEIs’ landing pages with poor accessibility, the number of errors increased even given the binding nature of the law. Obviously, there is still work to be done. Implications for practice are also discussed.

## Introduction

European higher education policies are strongly committed to inclusive education, enhancing opportunities for diverse learners [1, 2]. Since the Salamanca Statement [3], the main idea of inclusive education has remained the same: learning arrangements in which diverse students, including students with special educational needs or disabilities (SEND), can learn together with their peers. Consequently, diverse students should also have equal access to online study services. Accessibility means that “people with disabilities have access, on an equal basis with others, to the physical environment, transportation, information and communication technologies and systems (ICT) and other facilities and services” [1]. Accordingly, the landing page is often a first glance at the accessibility policy of a higher education institution (HEI) for applicants, students and stakeholders; HEIs with accessibility challenges immediately raise concerns about the accessibility of other webpages and online education as well. Equal participation and digital inclusion should be guaranteed to avoid building barriers and setting SEND students at a disadvantage compared to their nondisabled peers [4, 5]. Due to the COVID-19 pandemic, the role of online learning and digital services in HEIs has grown in a new way and to an unprecedented scale, embracing an even wider range of students.

The European Union (EU) has developed various policies and initiatives in an attempt to evolve towards a more equal society and strengthen Europe’s competitiveness by increasing digital skills in the workforce. Inaccessible digital services and the increasing pace of technological changes may expand the number of people excluded not only from education but from labour markets as well (Czaja & Urbaniec, 2019; Fichten et al., 2020). To promote equal possibilities for digital services, the European Commission announced the Digital Agenda for Europe [7], in 2010, and the Accessibility Directive [Directive [EU] 8/2102] entered into force in the EU in 2016. The Directive implies that public sector bodies must regularly evaluate the accessibility of their web services and provide Web Accessibility Statements [Directive [EU] 8/2102].

Turning from the European standpoint to the national scale, Act 306/2019 on the Provision of Digital Services [9], was enacted to implement the Accessibility Directive in Finland. Finland is known as a country of high technology and high-quality education. For example, Finland has been the leading country, in terms of digital performance and public digital services, among the 27 EU countries [10]. This sets high expectations regarding the quality and accessibility of the digital services of Finnish HEIs because they are public-sector bodies.

To contribute to the implementation of accessible online services, we conducted an empirical study on the accessibility of Finnish HEIs’ landing pages. Of the potentially tens of thousands of websites under a single HEI’s domain, the landing page is the most representative page and the one most frequently analysed in web accessibility studies [11–13]. Indeed, landing pages are a “parade door” or “main entrance” to other pages and often receive the most attention from visitors, web designers, and content developers [14].

The main research question of this study is as follows: *How accessible are Finnish HEIs’ landing pages?* The question is addressed through the following sub-questions:

- 1. What kinds of accessibility errors can be found, and how are they are revealed by two different automated test tools?
- 2. How has the accessibility of Finnish HEIs’ landing pages changed with the binding nature of the legislation?
- 3. What kind of accessibility profiles can be found among Finnish HEIs?

To answer the questions, we examined Finnish HEIs’ landing pages’ accessibility using two automated test tools. The data collection for this research was conducted before and after the new accessibility legislation for public-sector bodies, including HEIs. Also, the performance of these two automated test tools was analysed.

## Literature review

### Importance of accessibility

The World Health Organization [15] has estimated, in its 2011 World Disability Report, that 15% of the world’s population (one billion people) experience some form of disability and almost 200 million of them experience significant difficulties in functioning. According to the European Disability Strategy [1], in the EU, one in six people, or 80 million people, has a mild to severe disability. In addition, according to a variety of sources, it has been estimated that 10–15% of students in HEIs have some disabilities or special educational needs. In the EUROSTUDENT VII study, 15% of higher education students in Europe reported limitations in their studies due to a health impairment, most commonly either physical chronic diseases or mental health issues [16]. Overall, the diversity of students has been increasing. For example, in Finland, according to the results of national surveys, in 2016, 8,2% of higher education students reported having learning difficulties, with a disease or disability affecting their learning [17], and in 2021, the corresponding number was 14,2% [18], while reflecting the success of students in HE, Fichten, Olenik-Shemesh, Asuncion, Jorgensen and Colwell [5] find many reasons for the growing numbers of graduating students with disabilities, for example, the increased recognition of the abilities of students with disabilities; a redefinition of disability, including students other than those with mobility and sensory disabilities, and the de-medicalisation of disability in the HE context. Finally, they underline the supportive role of the increased presence of accessible ICTs within HE.

The foregoing numbers emphasise the importance of accessibility as a leading principle while developing digital services, including websites and learning environments. Notably, resent reviews emphasise two facets of online education: considering the characteristics of online students and applying evidence-based pedagogical practices [19, 20]. In addition to people with disabilities, accessibility-focussed research and development benefits everyone, especially those who have contextual constraints related to, for example, network connections, equipment, ambient sound or lighting conditions [21]. Therefore, applying web accessibility guidelines also seems to provide benefits to nondisabled users [22].

### Typical criteria for web accessibility

According to the World Wide Web Consortium (W3C) [23], web accessibility is “essential for developers and organizations that want to create high quality websites and web tools, and not exclude people from using their products and services.” The standard for the development of user interface accessibility defines accessibility as the ‘extent to which products, systems, services, environments, and facilities can be used by people from a population with the widest range of user needs, characteristics, and capabilities to achieve identified goals in identified contexts of use’ [24]. This definition also includes the use of assistive technologies. Overall, web accessibility describes the level to which a website can be used by as many people as possible [25].

The accessibility of websites is typically determined and tested using the Web Content Accessibility Guidelines (WCAG) criteria. Throughout this paper, when referring to the accessibility of websites, the term is used to determine assessment and compliance with WCAG (2.1). They are technical standards developed by W3C [26], and they provide design principles, recommendations and guidance for making web content more accessible to people with disabilities. The current version of the WCAG is 2.1, and the WCAG have been used as the basis for accessibility requirements in the legislation of many countries, such as the UK, the USA and Finland.

The WCAG include four principles that form the foundation of web accessibility. Thirteen WCAG design guidelines and 78 success criteria were based on them. The main structure of the WCAG is as follows:

- 1. Perceivable: Information and user interface components are presentable to users (text alternatives, time-based media, adaptable, distinguishable);
- 2. Operable: User interface components and navigation are operable (keyboard accessible, enough time, not causing seizures and physical reactions, navigable, input modalities);
- 3. Understandable: Information and the operation of the user interface are understandable (readable, predictable, input assistance);
- 4. Robust: Content is robust enough to be interpreted reliably by a wide variety of user agents, including assistive technologies (compatible).

All guidelines have testable success criteria at three levels of conformance: A (lowest), AA, and AAA (highest). For EU public websites, an AA level of conformance is required (Directive [EU] 2016/2102). Therefore, Finnish HEIs are also required to follow the AA level of the WCAG 2.1 criteria, with only one exception: captions are not required for live audio content in synchronised media (1.2.4 Captions (Live)).

### How to test the accessibility of websites

There are various ways of evaluating the accessibility of web pages, including expert evaluation, end-user testing, consigliere evaluation and automated testing tools [27, 28]. Overall, these various methods complement one another. Because the manual testing of web accessibility guidelines requirements may be difficult and burdensome, it is critical to have proper tools to assist in this [29]. Also, Paternò, Pulina, Santoro, Gappa, and Mohamad [30] state that the structured evaluation of public websites required by the European Directive can only be achieved with automatic support.

Automated test tools are widely used in evaluating the accessibility of web sites [31]. For example, Pribeanu [32] suggests a pragmatic strategy for accessibility evaluation at the national level, starting with the evaluation of the landing page of all websites using an accessibility evaluation tool to limit the workload. Many automated test tools are free extensions of web browsers and compare the accessibility of a page, for example, to the WCAG criteria. Automated tests are fast and easy to use and can also evaluate pages that are not published online. The tests describe accessibility issues and their effects and provide guidance on how to fix the problems; hence, the tester need not be an expert. There are several automated test tools. Previous research has found evidence that there are significant differences between the results obtained with various accessibility evaluation tools and suggests using more than one tool to increase confidence in results, e.g. Padure and Pribeanu [33].

On the other hand, the ability of automated test tools to identify problems is limited. A comparison of ten popular website accessibility tests in 2017 found 37% of problems (errors and warnings) at best and 17% at worst [34]. Automated test tools cannot detect all types of WCAG accessibility errors [31]. As a self-evident example, automated tests usually cannot detect how understandable the content or alternative texts are. Therefore, it is recommended to use more than one test method. Combining automated tools with manual checks, automated tests and user testing is an effective way to evaluate accessibility [34].

### Accessibility of HEIs’ websites

According to previous studies, there are significant accessibility challenges at HEIs’ landing pages; they have become more inaccessible, and their content has increased in complexity [14]. The major errors include low-contrast text and images; links without visible text; document file issues, such as inaccessible uploaded document files; missing alternative texts for images, as well as buttons, and a lack of navigation information (see [11, 12, 27, 35–39]). Furthermore, an analysis of 25 selected studies of educational websites’ accessibility showed that none of the sites were flawless [40]. An evaluation of top-ranking universities’ website accessibility found no significant improvements between 2005 and 2015 [14].

Also, the accessibility of Finnish HEIs’ websites has been evaluated several times [41, 42]. In 2008 and 2009, 59 landing pages of Finnish HEIs were evaluated. None of the pages were fully accessible. The most common problem was the lack of a text alternative for non-text content, such as images, and this occurred on all tested pages [41]. In another study, conducted in 2012, the accessibility of seven Finnish HEIs’ selected web pages was evaluated. Three HEIs’ sites reached more than 50% of the points available from the testing, two sites were hardly accessible and two sites were below the acceptable accessibility level. As a general assessment, the authors stated that pages of Finnish HEIs “were not bad” as compared to the pages of similar HEIs in various other countries [42].

### Summary

The diversity of higher education students, including students with disabilities, has been increasing. Still, the digital environments and services of HEIs’ should be accessible to all. The new European policy support this idea with new legislation. Web accessibility describes the level to which a website can be used by as many people as possible. The accessibility of websites is typically determined and tested using the WCAG criteria and principles (perceivable, operable, understandable and robust) and automated test tools. The test tools describe accessibility issues and provide guidance on how to fix the problems. Previous research has found evidence that there are significant differences between the results obtained with various accessibility evaluation tools. Research suggests using more than one tool to increase confidence in the results. According to previous studies, there are significant accessibility challenges at HEI’s landing pages. According to Finnish studies, none of the HEIs’ landing pages were fully accessible. The most common problem was the lack of text alternatives for non-text content, such as images, and this occurred on all tested pages.

## Methods

In this research, we analysed the accessibility of the landing pages of all Finnish HEIs (*N* = 38). The context of the study is the Finnish higher education system, which consists of universities and universities of applied sciences (UASs). Universities conduct scientific research and provide education based on it. Universities, offering higher scientific and artistic education, award bachelor’s and master’s degrees, as well as postgraduate degrees, i.e. licentiate and doctoral degrees. In comparison, UASs provide more practical education that aims to respond to the needs of the labour market. They mainly award UAS bachelor’s degrees but also some UAS master’s degrees [43].

### Data collection

The database of the Finnish Ministry of Education and Culture [43] was used as an information source to list all HEIs and their Finnish landing pages. Automated test tools were selected because they are available to everyone (including content providers and administrators), free of charge, fast and easy to use and do not require any special skills. A combination of two tools, Siteimprove and WAVE Web Accessibility Evaluation Tool, was chosen because, as noted in Sect. 2.3; previous studies have revealed that these tools emphasise different success criteria of the WCAG, as well as other factors affecting the accessibility of online content (e.g. the validity of the HTML). Therefore, the test results of these tools are non-identical. Both tools have been used for testing HEIs’ websites in previous studies. Correspondingly, both tools were mentioned among 25 selected studies on testing the accessibility of educational websites, and WAVE was one of the most popular tools [40].

The Siteimprove and WAVE test reports specify errors and warnings/alerts. In WAVE, errors are problems that must be fixed, and warnings are other elements that should be checked manually [44]. In Siteimprove, errors are failures to meet the WCAG success criteria, and warnings are failures to meet the best practices of the WCAG [45]. In this paper, we only consider errors to focus on the most serious accessibility issues. However, all test data (including Siteimprove’s review and warnings and WAVE’s features and alerts, structural elements, and ARIA) should be used to develop accessibility [46].

**Fig. 1:**
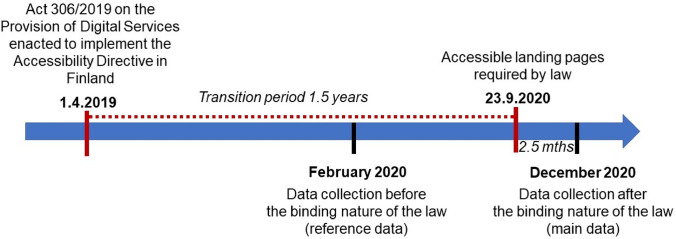
The timing of the accessibility of web pages, the Act on the Provision of Digital Services and the data collections of the current study

National legislation enacted to implement the Accessibility Directive in Finland in April 2019 was followed by a 1.5-year transition period before the law became binding. The first data (reference data) collection was conducted during the transition period in February 2020 (main issues introduced in [47]). The second data (main data) of this study were collected in December 2020, 2.5 months after the regulations related to the accessibility of websites came into force. These two data were compared to determine how the accessibility of Finnish HEIs’ landing pages has changed with the binding nature of the legislation. In this article, we name the first data the reference data and the second data the main data due to the more extensive analysis they receive. See Fig. 1 for an overview.

Both tools and data collections tested the A and AA conformance levels of the WCAG success criteria, but a different method was used for the data collection. First, the reference data were collected manually using the Chrome web browser (Chromium Version 80.0.3987) in February 2020. Two researchers performed tests manually using Siteimprove (Version 126) and WAVE (Version 3.0.4) add-ons and collected screenshots of the test results. The test results were then manually compiled into a table.

However, with the main data collection, Robot Framework automation was used to reduce the workload and minimise the variables potentially affecting the results. In other words, automation was used to ensure that data were collected in the shortest possible timeframe to avoid interference with browsers’ or automated test tools’ versions, as well as to minimise the changes in the content of the landing pages. The main data collection was carried out by automating the Chrome web browser (Chromium Version 81.0.4044.122), together with Selenium browser automation, image recognition based on desktop automation (PyAutoGUI) and Robot Framework automation with custom Python extensions in December 2020. Both screenshots and machine-readable summaries of the test results of the Siteimprove (Version 126) and WAVE (Version 3.1.2) add-ons were collected via automation. It took about 1 h and 45 min for the automation to collect or update the data at a time. Each HEI’s landing page was tested by both Siteimprove and WAVE within a 2-min timeframe.

Regarding data collection, the Chrome web browser was selected for both data collections because, at the point of testing, it was the most popular web browser across platforms (desktop, tablet and mobile). According to W3Counter Global web stats [48], Chrome’s market share was 58% in February 2020 and 65% in December 2020.

### Data analysis

The main research question, regarding how accessible Finnish HEIs’ landing pages are, is answered via three sub-questions. Different data and methods were used in the separate research questions (see Table 1).

**Table 1:** Research questions, data collection and methods used

The main research question is as follows: how accessible are Finnish HEIs’ landing pages?			
Sub-questions	Data collection period	Data collection tools	Methods
1.1. What kinds of accessibility errors can be found, and how are they revealed by two different automated test tools?	12/2020	Siteimprove, WAVE	Descriptive methods: frequencies, Wilcoxon Signed Rank Test
1.2. How has the accessibility of Finnish HEIs’ landing pages changed with the binding nature of the legislation?	02/2020 12/2020	Reorganised data (Siteimprove and WAVE)	Comparing two data: Wilcoxon Signed Rank Test
1.3. What kind of accessibility profiles can be found among Finnish HEIs?	12/2020	Reorganised data (Siteimprove and WAVE)	Hierarchical cluster analysis, Kruskal–Wallis Test, T-test (2-tailed)

As is already known, Siteimprove and WAVE mainly find the same accessibility issues based on the WCAG criteria, but they detect different amounts of them. To eliminate the quantitative bias produced by duplication, a new variable was created by comparing the error rates produced by different tools one criterion at a time and selecting a larger number of errors. This made the actual number of errors visible and made it possible to compare the changes at the level of error types. The reorganised data were used to answer Sub-questions 1.2 and 1.3.

A descriptive statistical analysis and the Wilcoxon Signed Rank Test were used to find answers to Sub-questions 1.1 and 1.2. Hierarchical cluster analysis and the Kruskal–Wallis test were used to answer Sub-question 1.3. Table 1 describes the data and methods used to study each research question.

In the following sections, the results are presented in the order of the research questions (Table 1).

## How accessible are Finnish HEIs’ landing pages?

### Remarkable differences between institutions

The number of accessibility errors on the landing pages of Finnish HEIs (*N* = 38) varied remarkably between HEIs and the two testing tools. To begin, in Siteimprove’s report, 29% of HEIs had fewer than 20 errors, while 32% had more than 100 errors on their landing pages. In WAVE’s report, the corresponding numbers were 13% of HEIs having fewer than 20 errors and 47% having more than 100 errors on their landing pages. Siteimprove reported an average of 87 errors per institution (minimum 0, median 50, maximum 685). WAVE reported an average of 65 errors per institution (minimum 0, median 21, maximum 704). WAVE found a total of 2,471 errors, while Siteimprove reported 3,297 errors.

When considering the HEIs, the fewest errors were found on the landing pages of Laurea UAS (Siteimprove 3, WAVE 1). The most errors were found on the landing page of Åbo Akademi University (Siteimprove 685, WAVE 704). Figure 6 in Appendix 1 illustrates the number of accessibility errors found by WAVE and Siteimprove on HEIs’ landing pages.

As Fig. 6 in Appendix 1 shows, the different testing tools found different numbers of errors (differences are discussed in Sect. 4.2). However, it is important to take a closer look at the types of errors.

### Types and frequency of accessibility errors on landing pages

The automated test tools found different numbers and types of accessibility errors on the landing pages. The Wilcoxon test was used to compare the number of accessibility errors found by both tools. The difference between the total number of errors found by the tools was significant (*p* = 0.043). As can be seen from Table 2 below, there were significant differences between Siteimprove and WAVE within eight of the 15 WCAG criteria: Siteimprove reported more errors on six criteria, whereas WAVE reported more errors on two criteria. Table 2 presents the types, frequencies and significance levels of the errors.

**Table 2:** Accessibility errors of the main data collection by Siteimprove and WAVE

WCAG criteria	Description	WCAG criteria level	Errors by Siteimprove	Errors by Wave	*p*
1. Perceivable	Information and user interface components must be presentable to users in ways they can perceive				
1.1.1 Non-text content	All non-text content (e.g. images) has a text alternative that has an equivalent purpose	A	321*	**371**	**0.136**
1.3.1 Info and relationships	Information, structure and relationships conveyed through presentation can be programmatically determined or are available in text (e.g. headings are marked as headings)	A	250	89	0.003*
1.4.1 Use of colour	Colour is not used as the only visual means of conveying information, indicating an action, prompting a response or distinguishing a visual element	A	172	0	0.001*
1.4.3 Contrast (minimum)	The visual presentation of text and images of text has a contrast ratio of at least 4.5:1	AA	**798**	**1431**	**0.195**
2. Operable	User interface components and navigation must be operable				
2.1.1 Keyboard	All functionality of the content is operable through a keyboard interface, without requiring specific timings for individual keystrokes	A	0	6	0.317
2.4.1 Bypass block	A mechanism is available to bypass blocks of content that are repeated on multiple web pages	A	0	20	0.024*
2.4.2 Page titled	Web pages have titles that describe the page’s topic or purpose	A	2	0	0.157
2.4.4 Link purpose (in context)	The purpose of each link can be determined from the link text alone or from the link text together with its programmatically determined link context	A	**954**	**391**	**0.061**
2.4.6 Headings and labels	Headings and labels describe topic or purpose	AA	0	87	0.000**
2.4.7 Focus visible	Any keyboard-operable user interface has a mode of operation in which the keyboard focus indicator is visible	AA	345	0	0.000**
3. Understand-able	Information and the operation of user interface must be understandable				
3.1.1 Language of page	The default human language of each web page can be programmatically determined	A	1	1	1.000
3.2.2 Predictable on input	Changing the setting of any user interface component does not automatically cause a change of context (e.g. opening a pop-up window unexpectedly), unless the user has been advised	A	10	0	0.004*
3.3.2 Labels or instructions	Labels or instructions are provided when content requires user input (e.g. forms)	A	33	67	0.150
4. Robust	Content must be robust enough that it can be interpreted by a wide variety of user agents, including assistive technologies				
4.1.1 Parsing	In content implemented using markup languages, elements have complete start and end tags, elements are nested according to their specifications, elements do not contain duplicate attributes and any IDs are unique	A	133	0	0.003*
4.1.2 Name, role, value	For all user interface components (including form elements, links and components generated by scripts), the name and role can be programmatically determined. States, properties, and values that can be set by the user can be set programmatically. Notification of changes to these items is available to user agents, including assistive technologies	A	278	8	0.000**
Total number of errors			3,297	2,471	

*N* = 38, *p < 0.05; **p < 0.001

^*^The most common errors are emphasised with bold

To begin with, it is notable that most of the errors reported were at the lowest conformance level (A). Thus, even if the tools found different numbers of errors, the three most common accessibility issues were still the same but in different order (see the order of frequency in Table 2):

- The colour contrast of the text and background was insufficient. WAVE reported inadequate contrasts in more detail, with 58% (1,431 of the 2,471) of the errors on HEIs’ landing pages involving too-low colour contrasts;
- The link’s purpose could not be determined from the link text;
- Text alternatives describing non-text content in the form of text were missing.

To gain a deeper understanding of the characteristics and results of the different tools in terms of the WCAG, the total numbers of errors based on the main data collection were compared.

**Fig. 2:**
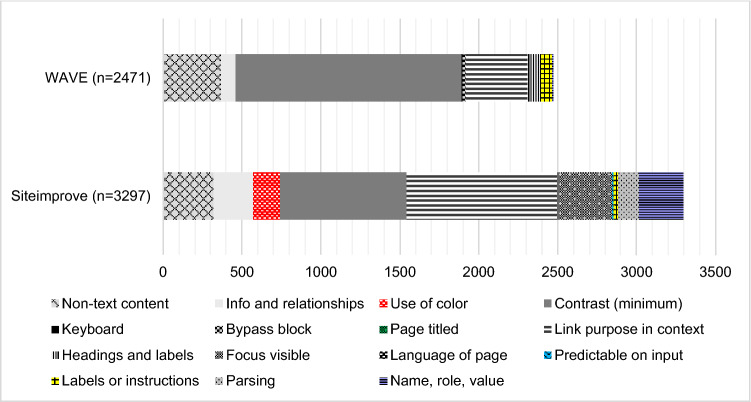
Numbers of errors found by WAVE and Siteimprove on HEIs’ landing pages

Most of the errors reported by WAVE were contrast errors, while Siteimprove found most issues related to link purpose in context (see Fig. 2). Figure 2 illustrates the differences in the numbers of error types found by Siteimprove and WAVE. It is notable that within several types of WCAG criteria, one tool found zero errors, whereas the other found tens or even hundreds of the same errors.

Overall, these results indicate that it is recommended to use more than one automated test tool because their reports are not identical.

### How has the accessibility of Finnish HEIs’ landing pages changed with the binding nature of the act on the provision of digital services?

To evaluate the impact of the new legislation, the data collected before and after the law were compared. After the Act became binding, the total number of accessibility errors decreased by one-fifth (from 5,506 to 4,504). In more than half of the HEIs (*n* = 20), the number of accessibility errors decreased, whereas it had, surprisingly, increased in 14 HEIs. Four HEIs remained the same in this regard. Before the law, there were an average of 145 errors per institution (minimum 5, maximum 844), and after the law, there were an average of 119 errors (minimum 3, maximum 1,165). However, the difference between the two data collections was not significant (*p* = 0.050).

**Fig. 3:**
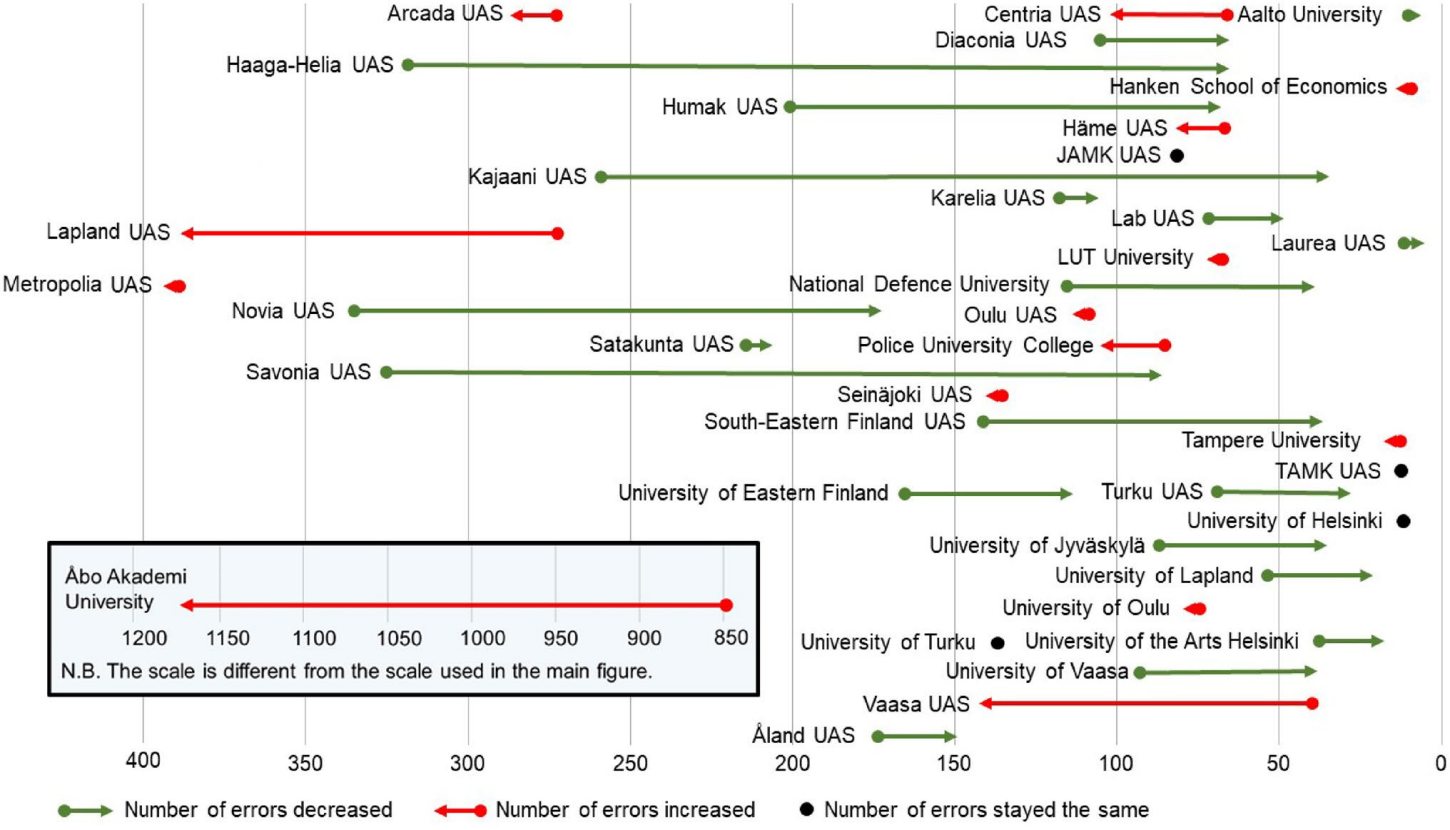
Changes in the number of errors on HEIs’ landing pages

Overall, there were more large improvements in error rates than large deteriorations. Those HEIs that had few errors before the law became binding also had few errors after it: Laurea UAS, University of Helsinki, Aalto University, Tampere UAS, and Tampere University. When comparing the HEIs, Åbo Akademi University distinguishes itself in terms of the number of errors. To begin with, it had the most accessibility errors on its landing page, both before and after the law. Second, the number of errors on its landing page also increased the most, by a considerable amount, as compared to other HEIs. Third, the errors made by Åbo Akademi University’s landing pages were one-fourth of all HEIs’ errors on the second data collection. It is notable that the majority of Åbo Akademi University’s errors were contrast errors. Figure 3 illustrates the direction of the development and the extent of the change in the number of errors on HEIs’ landing pages.

**Fig. 4:**
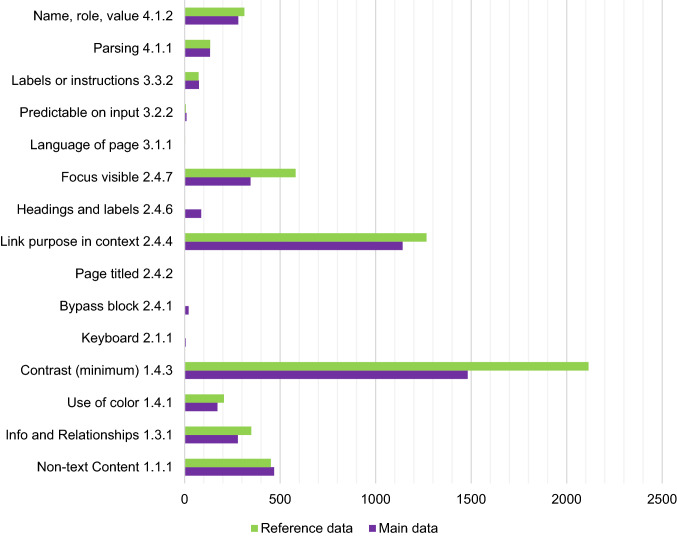
Accessibility errors found on HEIs’ landing pages on reference and main data collection

Considering all 15 types of accessibility errors found, the number had increased in six types, decreased in eight types and remained the same in one (see Fig. 4). Surprisingly, there was one new error type (keyboard) in the second data collection. It is also noteworthy that the most common errors in the first data collection remained the same in the second data collection.

A Wilcoxon test was used to compare the number of accessibility errors in the data collected before and after the requirements of the law came into effect to evaluate how the Act becoming binding has affected Finnish HEIs’ landing pages. The results indicate significant changes in three of the 15 accessibility criteria. First, the number of contrast errors decreased significantly (*p* = 0.006). Interestingly, the number of errors increased in two criteria: bypass blocks (*p* = 0.034) and headings and labels (*p* = 0.000).

### What kind of accessibility profiles can be found among Finnish HEIs?

The accessibility profiles of HEIs’ landing pages were compared using a hierarchical cluster analysis and the Kruskal–Wallis test. Profiles were based on the level of four WCAG principles (perceivable, operable, understandable and robust), not on individual error types. The differences between the two clusters were statistically significant within all four WCAG principles (see Table 3).

**Table 3:** Statistical significance levels of the differences in WCAG principles

	Perceivable	Operable	Understandable	Robust
Test statistic	22,250	18,200	10,249	6,448
Asymptotic sig. (two-sided test)	0.000**	0.000**	0.001*	0.011*

*Df* = 1, *p < 0.05; **p < 0.001

**Fig.5:**
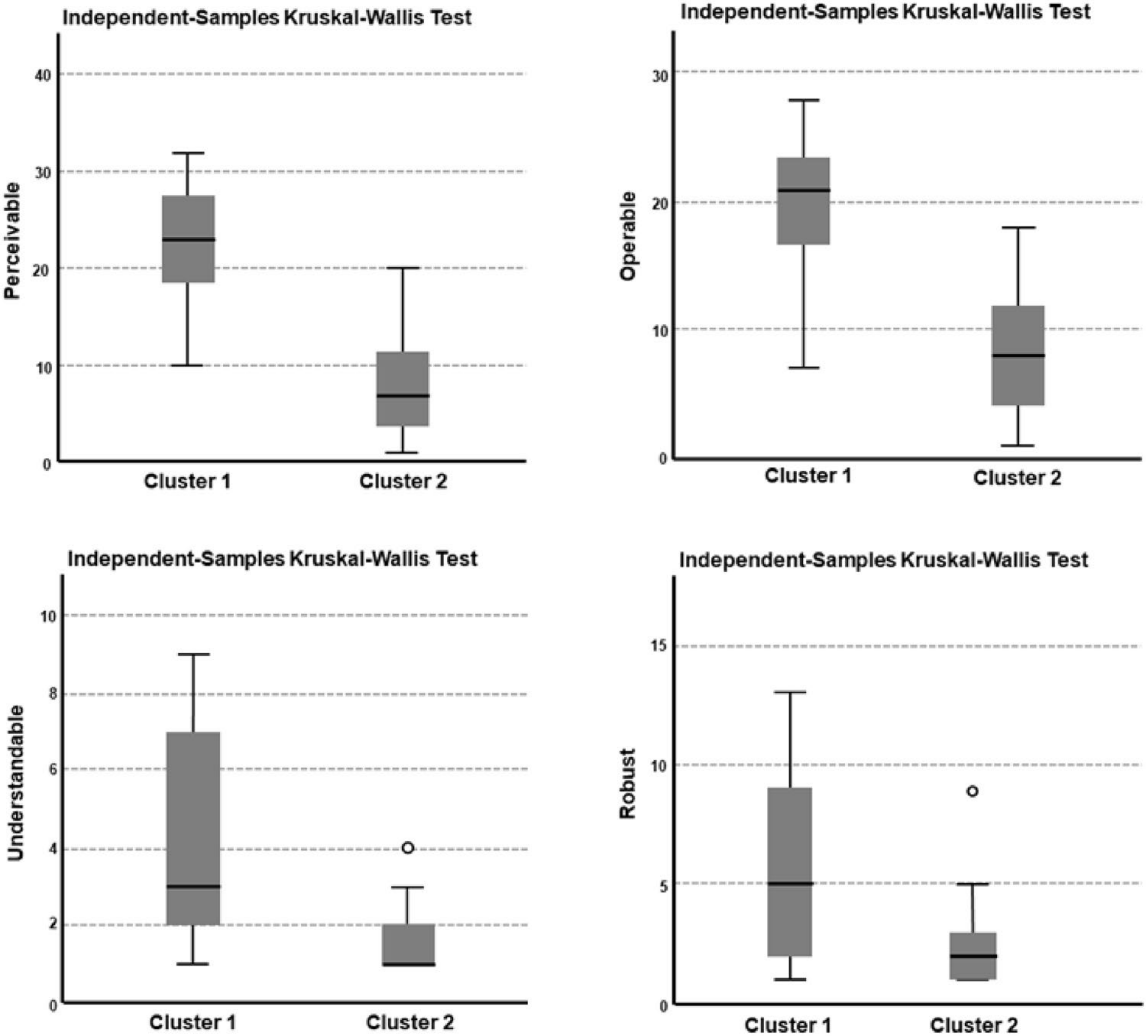
Box plots of the WCAG principles (perceivable, operable, understandable and robust) for Clusters 1 and 2

**Fig. 6:**
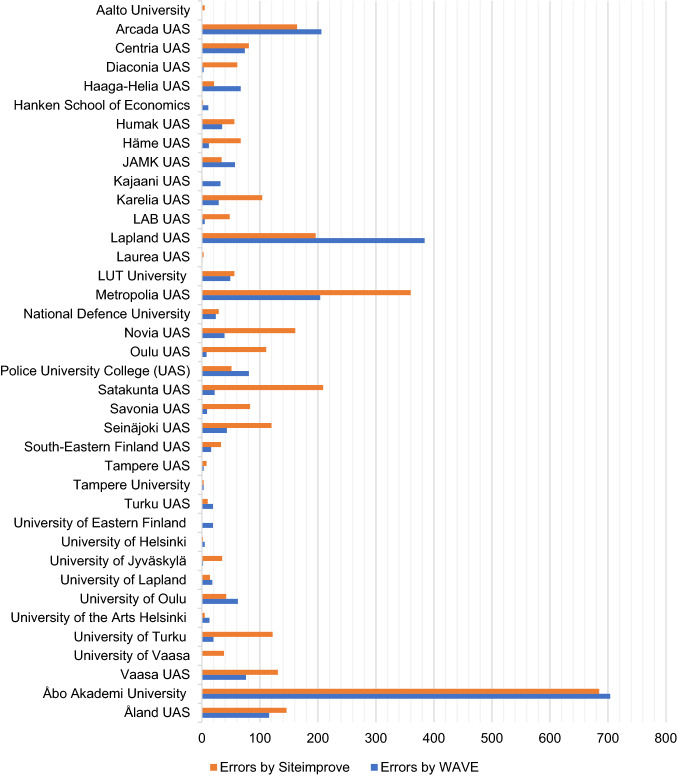
Number of accessibility errors found by WAVE and Siteimprove on HEIs’ landing pages

However, the categories of perceivable and operable exhibited the largest differences. Figure 5 illustrates the differences between the clusters.

As a result of the analysis, the HEIs were divided into two clusters, with 19 HEIs in each (see Table 4 in Appendix 2). The accessibility of Cluster 1’s landing pages was poor in terms of all four principles, whereas the accessibility of Cluster 2’s landing pages was good in terms of all four WCAG principles.

**Table 4:** Two clusters of HEIs based on the accessibility of their landing pages [61]

Cluster 1	Staff (N)	Cluster 2	Staff (N)
Arcada UAS	160	Aalto University	4,217
Centria UAS	296	Diaconia UAS	237
Haaga-Helia UAS	613	Hanken School of Economics (University)	275
Häme UAS	599	Humak UAS	131
JAMK UAS	657	LAB UAS	474
Kajaani UAS	216	Laurea UAS	549
Karelia UAS	258	LUT University	1,028
Lapland UAS	420	National Defence University (University)	250*
Metropolia UAS	895	South-Eastern Finland UAS	793
Novia UAS	261	Tampere UAS	749
Oulu UAS	426	Tampere University	3,668
Police University College (UAS)	200*	Turku UAS	688
Satakunta UAS	366	University of Eastern Finland	2,602
Savonia UAS	493	University of Helsinki	7,388
Seinäjoki UAS	326	University of Jyväskylä	2,560
University of Turku	3,265	University of Lapland	590
Vaasa UAS	498	University of Oulu	2,977
Åbo Akademi University	1,145	University of the Arts Helsinki	751
Åland UAS	59**	University of Vaasa	498

^*^Information retrieved from institution’s webpage

^**^Information retrieved from personal communication

Interestingly, most of the science universities (university) were in Cluster 2, while universities of applied sciences (UASs) were divided between clusters (see Table 4 in Appendix 2). When comparing the number of staff in the different clusters with a t-test (two-tailed), HEIs with more accessibility issues on their landing pages were, on average, smaller organizations than in the “advanced” cluster (Cluster 2). The difference between the two clusters was significant (*p* = 0.038).

## Discussion

### Discussion of major findings

The main research question in this study sought to determine the accessibility of Finnish HEIs’ landing pages. The study concerned all Finnish HEIs. As discussed in Sect. 1, landing pages are often seen as the “main entrance” to other pages and relate to the accessibility issues on other pages. In addition, the changes in accessibility as compared to the pre-statutory period were examined.

As a main result, it was remarkable that the majority of Finnish HEIs’ landing pages were still not accessible, even after the binding nature of the Act. Also, none of the landing pages were fully accessible, and the differences between institutions were notable. When comparing the institutions in terms of WCAG accessibility principles, two clusters were found: one recognised as having “good” accessibility and the other recognised as having “poor” accessibility in terms of all four principles. Still, in the big picture, when comparing the accessibility of landing pages in different periods, the direction was the correct one in most of the HEIs: towards better accessibility. In the following section, the results will be examined in more detail.

#### Typical types of errors revealed by the two tools

As indicated in previous studies and confirmed in this study, different test tools find different types^1^ and numbers of errors. The most general error types were the same for Siteimprove and WAVE: *contrasts, link purpose in context* and the lack of *text alternatives describing non-text content*, such as images. These were among the most common errors in previous studies (see [14, 36, 40]).

Based on previous studies, the wide extent of *insufficient colour contrast* between the text and background (including text on images, buttons, and icons) was rather foreseeable [40, 46]. It is notable that insufficient contrast concerns a wide range of website users. *Insufficient colour contrast* can make it difficult or even impossible to read the website and access the associated content. Colour contrast is of considerable importance to people with impaired vision, low contrast sensitivity or colour blindness. It also concerns mobile users, who view websites on the small screens of mobile devices in varying lighting conditions, such as sunlight and glare. However, at a technical level, colour contrast is easy to check (e.g. with colour contrast analysers) and fix.

Determining the *purpose of a link* from the link text refers to generic link texts, such as “Read more.” A user with a screen reader, an assistive technology that renders text and image content as speech or braille output, typically lists the links in alphabetical order when searching for information. In this case, a list of identical “Read more” links will not help find the desired information. As Scanlon et al. [38], state, users should know exactly where a link will lead them. This error likely indicates a lack of knowledge on the part of web content providers, and it is easy to solve the problem by instructing them regarding how to create accessible links.

The lack of *text alternatives describing non-text content* as text hinders, for example, the access of visually impaired readers to information also makes it more difficult for search engines to find the content. It was the most common problem in the Finnish HEIs’ landing pages in a previous study [41]. It is notable that most automated tests only note whether the alternative text is missing, not whether this text is uninformative or not understandable. This problem should be fixed by guiding web content providers to write appropriate text alternatives and, when necessary, mark images as decorative.

WAVE’s strength was detecting minimum *contrasts,* and Siteimprove’s was finding *link purpose in context* errors. It is notable that WAVE did not find any *focus visible* errors, whereas Siteimprove did not find any errors concerning *headings and labels*. These results indicate that it is recommended to use more than one automated test tool, as their reports are not identical.

Surprisingly, there were many accessibility issues, even for the lowest conformance level (A), which cause problems for a wide range of users. Two of the three most common accessibility errors occurred at Level A, and one occurred on Level AA (see Table 2 for the conformance levels of the errors).

Typically, issues found in this research were the same as in previous studies of the accessibility of HEIs’ websites, including Finnish HEIs’ websites (see Sect. 2.4). However, it is important to consider all accessibility issues, as they can prevent access to content entirely.

#### Comparing HEIs

The accessibility of HEIs’ landing pages varied greatly. In terms of the number of accessibility errors, a few HEIs had almost flawless pages, but on average, there were many problems. Several HEIs’ landing pages had hundreds of issues, and one HEI had even well over a thousand errors. The average number of accessibility errors using the combination of Siteimprove and WAVE was 119 per institution (minimum 3, maximum 1,165).

The average number of errors was rather high as compared to previous studies. To discuss this point in more detail, the average number of errors found by WAVE was 65, which is comparable to other studies that used only WAVE. The number of WAVE errors was high when compared to the average 24 accessibility errors on US university websites tested with WAVE [46]. Also, the WebAIM Million report’s information of 51 errors per page for educational organisations, as well as the 51 errors per page on the Internet’s one million favourite websites, is also exceeded in this study [49].

#### Did the law make any difference?

With the support of legislation, the accessibility of HEIs’ landing pages seems to have improved. When considering the error types, the number of *contrast* errors diminished significantly after the law. In contrast, there were more errors in *bypass blocks* and *headings and labels*. Surprisingly, one new error type *(keyboard)* was found. However, it was notable that HEIs with good accessibility were the same before and after the law became binding, while institutions with poor accessibility remained poor.

The cluster analysis pointed out that the differences in terms of accessibility between the two clusters were clear. There were significant differences related to all four WCAG principles. Most universities were located in the “better” cluster, whereas UASs were divided into both clusters. The HEIs in the “worse” cluster have plenty of work to do to improve the accessibility of their landing pages. Clustering illustrated that landing pages’ accessibility (on the level of the four WCAG principles) was connected to the number of staff. It is notable that UASs are typically smaller institutions than universities. The differences between HEIs may suggest a lack of clear national guidelines and supervision.

The Act on the Provision of Digital Services came into force in Finland on April 1, 2019. There was a 1.5-year transition period for institutions to make their websites accessible. Therefore, HEIs should have had enough time to improve their accessibility. Even 2.5 months after the law became binding, the number of errors was high on many landing pages. It seems that sufficient understanding, commitment or resources may not have been allocated. Also, Fichten et al. [5] point out that even if legislation is probably an essential prerequisite for accessibility improvements, it is not enough on its own: practitioners require instructions on how to translate the rules into practice.

### Implications for practice

Vollenwyder et al. [50] have collected, from previous studies, some potentially harmful conceptions of web accessibility based on insufficient knowledge. For example, web accessibility compromises aesthetics and technologically advanced solutions, being merely the developers’ responsibility, only involving people with visual impairments or being either free of charge or very expensive. Nevertheless, these assumptions do not agree with the research findings: accessibility enhances websites’ performance and usability for everyone, without impacting visual appeal.

The following practical implications, based on lessons learned during this research, could be useful for institutions that are willing to improve the accessibility of their webpages.

#### Resources

Testing and improving accessibility are continuous processes. The level of accessibility may change radically as the result of new or updated content, software program, platform or browser version or an organisation’s a website renewal. It may be a question of resources in terms of working hours. However, testing and improving accessibility are not expensive; all the tools needed are available free of charge.

#### Division of work

As described above, different professionals are responsible for different kinds of errors. Some errors are more technical in nature, while others are more related to the way content is shared and presented. All staff adding or modifying the content or technology related to webpages must recognise their own roles and know their responsibilities. In a broader context, King et al. [51] argue that experts require a broad understanding of accessibility issues (e.g. knowledge of both disabilities and ICT) in HEIs. They also suggest that experts should work cooperatively to share ideas and practices.

#### Using support of technology

Due to the huge number of content producers, technological support is needed. There are many accessibility testing tools available, and they are easy to use. While attempting to reach accessible webpages, it would not be realistic to suppose that everyone knows all the details of accessibility requirements. Service providers will likely continue developing more accessible solutions due to regulations affecting their business. If a more powerful approach or more systematic processes are needed, institutions can use compulsory elements, for example, to force the addition of alternative texts to images or captions to videos before publishing them.

#### Staff training

It is important to inform and train staff to develop accessible practices and avoid bias and lack of knowledge [52–55]. Accessible solutions are good and beneficial solutions for all, not only for people with visual impairments or some marginal groups. It is not a voluntary choice but, rather, a law-based, mandatory duty. Thus, it is important to learn how to produce accessible content or platforms and how to test them. For many accessibility errors, the fixes are not complicated but can be done by training the content providers. Taking care of accessibility should be a natural part of everyday work and a factor to evaluate when new technology is acquired and mobilised.

#### Cooperation and institutional policies

The best results are achieved by working together and clarifying responsibilities across an entire institution and all its stakeholders [51]. An individual can contribute to accessibility within their own duties, but there is a need to foster awareness, knowledge, guidance, skills, and leadership on all organisational levels to develop community action. It may be necessary to have institutional guidelines for some details, e.g. the content of web pages. Dynamic content, such as social media feeds that are not under the control of institutions, causes accessibility errors. Guidelines that concretise policies could also include institutional processes and responsibilities. Achieving accessibility is a cultural transformation that requires the commitment of the entire HEI community and the support of a cooperative network. According to Merchant et al. [56], the first step is to confront and identify the values that underpin higher education.

#### National policies

Higher educational institutions are rather autonomous in Finland. However, the Ministry of Education and Culture plans and implements higher education and science policy. It also prepares statutes, national budget proposals and government decisions concerning them, as well as steering the activities of the higher education system [57]. While considering the differences between the accessibility of HEIs’ landing pages, it is obvious that guidelines and supervision, at the national level, are needed. Moving towards more accessible higher education and HEIs is one of the major topics in Finnish higher education policy currently [58]. Taking care of the accessibility of webpages is one step towards that goal.

### Limitations

This research concentrated on the landing pages of all Finnish HEIs. Previous research stated that there is a connection between the accessibility of landing pages and other pages. Still, this research represents a fairly narrow sampling of the institutions’ web pages. Because the focus of accessible design may have been only on the landing page, a larger sampling would offer a broader view of accessibility.

The sampling was performed twice; this paints a picture of the accessibility of the landing pages in two separate moments. It is important to note that complete flawlessness can be temporary because websites are dynamic in nature, with, for example, changing banner images, social media streams and videos. For the same reason, the numbers of certain types of errors may be exaggerated.

The errors presented in this study were those measured by selected tools. The situation can be even more critical because automated testing tools do not reveal all accessibility issues, such as whether the videos are captioned. For more reliable results, the landing pages should have been tested with several web browsers, test tools and platforms, as well as via user testing.

## Conclusion

Online education and digital services in higher education have grown rapidly. The COVID-19 pandemic has forced even traditional face-to-face institutions to make online education and services available for all students. At the same time, new legislation related to the accessibility of public services makes it mandatory to offer accessible services and education to all students. New kinds of attitudes, knowledge and skills are needed, and such change requires leadership.

Accessibility evaluation for web pages is an ongoing process. Due to the dynamic nature of web pages, accessibility should be continuously monitored and tested. The present study is easy to replicate: both the testing tools and HEIs’ landing pages, in their current form, are available to anyone, and therefore, it is easy to analyse the current situation and track the changes in accessibility. Therefore, the study provides a good basis for Finnish HEIs to consider and evaluate the direction of their web pages’ accessibility.

Overall, a wide range of current European legislation supports equal opportunities and rights for all people, and there is more to come in terms of national legislation [Directive [EU] 59/882]. Still, slow progress on accessibility can be expected; Wattenberg [60] has already discussed the effect of legislative measures on the accessibility of online education, concluding that there has been no real improvement 10 years after the implementation of the Americans with Disabilities Act (ADA).

However, it is not only a question of law but also of inclusive practices and good education for all. If we accept using HEIs’ landing pages as an indicator of the accessibility of institutions’ web pages, institutions still have a great deal of work to do. As a final conclusion, it can be said that the direction is right, but the speed is too slow.

## Data Availability

Data can be accessed by contacting the authors.

## Appendix

See Fig. 6 and Table 4

## Footnotes

1 The names of the errors are in *italics*. See Table 2 for detailed descriptions.
